# CXCL14 promotes metastasis of non-small cell lung cancer through ACKR2-depended signaling pathway

**DOI:** 10.7150/ijbs.79438

**Published:** 2023-02-27

**Authors:** Tsung-Ming Chang, Yao-Chang Chiang, Chiang-Wen Lee, Chieh-Mo Lin, Mei-Ling Fang, Miao-Ching Chi, Ju-Fang Liu, Yu Ru Kou

**Affiliations:** 1 Department and Institute of Physiology, College of Medicine, National Yang Ming Chiao Tung University, Taipei 11221, Taiwan.; 2 Department of Nursing, Division of Basic Medical Sciences, and Chronic Diseases and Health Promotion Research Center, Chang Gung University of Science and Technology, Chiayi 61363, Taiwan.; 3 Research Center for Industry of Human Ecology and Research Center for Chinese Herbal Medicine, Chang Gung University of Science and Technology, Taoyuan 33303, Taiwan.; 4 Department of Safety Health and Environmental Engineering, Ming Chi University of Technology, New Taipei City 24301, Taiwan.; 5 Department of Orthopaedic Surgery, Chang Gung Memorial Hospital, Chiayi 61363, Taiwan.; 6 Division of Pulmonary and Critical Care Medicine, Chang Gung Memorial Hospital, Chiayi 61363, Taiwan.; 7 Graduate Institute of Clinical Medical Sciences, College of Medicine, Chang Gung University, Taoyuan 33302, Taiwan.; 8 Center for Environmental Toxin and Emerging-Contaminant Research, Cheng Shiu University, Kaohsiung 83347, Taiwan.; 9 Super Micro Research and Technology Center, Cheng Shiu University, Kaohsiung 83347, Taiwan.; 10 Department of Respiratory Care, Chang Gung University of Science and Technology, Chiayi 61363, Taiwan.; 11 School of Oral Hygiene, College of Oral Medicine, Taipei Medical University, Taipei 11031, Taiwan.; 12 Translational Medicine Center, Shin-Kong Wu Ho-Su Memorial Hospital, Taipei 11101, Taiwan.; 13 Department of Medical Research, China Medical University Hospital, China Medical University, Taichung 40402, Taiwan.; 14 Department of Medical Research, Hualien Tzu Chi Hospital, Buddhist Tzu Chi Medical Foundation, Hualien 97002, Taiwan.

**Keywords:** human lung cancer, CXCL14, ACKR2, metastasis, epithelial mesenchymal transition

## Abstract

**Background:** Lung cancer is a malignant tumor with metastatic potential. Chemokine ligand 14 (CXCL14) has been reported to be associated with different cancer cell migration and invasion. However, few studies have explored the function of CXCL14 and its specific receptor in lung cancer metastasis. This study aims to determine the mechanism of CXCL14-promoted cancer metastasis.

**Methods:** The expression of CXCL14, atypical chemokine receptor 2 (ACKR2), and epithelial mesenchymal transition (EMT) markers was evaluated by the public database of The Cancer Genome Atlas (TCGA) and Gene Expression Omnibus (GEO), Western blot, enzyme-linked immunosorbent assay (ELISA), quantitative real-time polymerase chain reaction (qPCR), immunohistochemistry (IHC), and immunofluorescence (IF). Migration and wound healing assays were used to observe the motility of cancer cells. A luciferase reporter assay was performed to analyze transcription factor activity. The metastasis of lung cancer cells was evaluated in an orthotopic model.

**Results:** We have presented that overexpression of CXCL14 and ACKR2 was observed in lung cancer datasets, human lung tumor sections, and lung cancer cells. Furthermore, the migration of CXCL14-promoted lung cancer cells was determined *in vitro* and *in vivo*. In particular, ACKR2 knockdown abolished CXCL14-induced cancer cell motility. Additionally, ACKR2 was involved in CXCL14-triggered phospholipase Cβ3 (PLCβ3), protein kinase Cα (PKCα), and proto-oncogene c-Src signaling pathway and subsequently upregulated nuclear factor κB (NF-κB) transcription activity leading to EMT and migration of lung cancer cells. These results indicated that the CXCL14/ACKR2 axis played an important role in lung cancer metastasis.

**Conclusion:** This study is the first to reveal the function of CXCL14 in promoting EMT and metastasis in lung cancer. As a specific receptor for CXCL14 in lung cancer, ACKR2 mediates CXCL14-induced signaling that leads to cell motility. Our findings can be used as a prognostic biomarker of lung cancer metastasis.

## Introduction

Lung cancer is one of the leading global causes of cancer mortality 1. Although chemotherapy and radiation therapy have contributed to improvements in lung cancer treatment, survival rates remain poor, overall survival rates at 5 years of patients with non-small cell lung cancer (NSCLC) and small cell lung cancer are 15% and 6%, respectively 2, 3. Metastasis progression plays a crucial role in advanced lung cancer, leading to a poor prognosis and low survival rates 4. Therefore, an understanding of the molecular mechanism of cancer cell metastasis has become a major subject of interest in lung cancer treatment 5.

C-X-C motif chemokine ligand 14 (CXCL14), known as breast and kidney-expressed chemokine (BRAK), is a member of the chemokine family and is responsible for the chemotactic effects on neutrophils 6. CXCL14 is generally expressed at high levels in numerous tissues such as the breast, kidney, lung, and skin 7. However, the mechanism of CXCL14 is attracting increasing attention because abnormal expression of CXCL14 could play an important role in several cancers. CXCL14 is ambiguous and disparate in different types of cancers. In head and neck squamous cell carcinoma and colorectal cancer, CXCL14 suppresses cancer cell migration and invasion 8. Furthermore, CXCL14 is overexpressed in the stromata of the breast and pancreas and prostate cancer 9-11. In addition, CXCL14 is associated with metastasis of lung cancer 12. Therefore, understanding the mechanisms responsible for CXCL14-promoted metastasis in lung cancer and identifying the key factors involved will identify new therapeutic targets.

Increasing evidence has indicated that CXCL14 can bind to the atypical chemokine receptor 2 (ACKR2) 13. ACKR2 is found in endothelial cells and leukocytes in the intestine, lungs, skin, and lymphoid organs 14. The abnormal expression of ACKR2 is associated with many human diseases, including hepatitis, psoriasis, and cancer 15-17. ACKR2 is overexpressed in Kaposi's sarcoma and primary effusion lymphoma cells 18. Moreover, CXCL14 binds to ACKR2 in cancer-associated fibroblasts (CAF) resulting in breast cancer metastasis 13, 19. Therefore, it may be worthwhile to target CXCL14/ACRK2-induced cancer metastasis in NSCLC.

Here, this study provides an overview of CXCL14 progressively upregulated along with the progression of NSCLC and associated with tumor metastasis. We also found that CXCL14 promotes the epithelial-mesenchymal transition (EMT) and subsequently improves cell migration by transactivating the ACKR2/PLC/PKC/c-Src signaling pathway. Knockdown of CXCL14 suppressed EMT production in NSCLC cells and attenuated cell migration ability. Importantly, CXCL14 overexpressed profoundly enhanced lung cancer cell metastasis. Our findings offer new insights into CXCL14 as a potential therapeutic target for cancer invasion and metastasis of lung cancer.

## Materials and Methods

### Analysis of public data sets of CXCL14 and ACKR2 in normal and NSCLC lung tissues

Lung cancer adenocarcinoma (LUAD) data sets from the meta-analysis were downloaded from Lung Cancer Explorer (LCE), which contains data sets of gene profiles of tumor and normal lung samples from The Cancer Genomics Atlas (TCGA) and other published literature. All data sets for the TCGA gene analysis were downloaded from UALCAN, which contains data sets of the gene profiles of tumor and normal lung samples obtained from TCGA. Transcriptome data were obtained from the GEO database.

### Materials

Primary antibodies specific for phospho-phospholipase Cβ3 (PLCβ3; Ser537, #2481), PLCβ3 (#14247), phospho-protein kinase Cα/βII (PKCα/βII; Thr638/641, #9375), PKCα (#2056), phospho-proto-oncogene c-Src (c-Src; Ser17, #5473), c-Src (#2109), phosphor-inhibitor of NF-κB α (IκBα; Ser32/36, #9246), phosphor-IκB kinase α/β (IKKα/β; Ser176/180, #2697), phospho-NF-κB p65 (Ser536, #3033), NF-κB p65 (#8242), and ZO-1 (#8193) were purchased from Cell Signaling Technology (Danvers, MA, USA). IκBα (ab32518) was purchased from Abcam (Cambridge, UK), and CXCL14 (GTX108431), ACKR2 (GTX21658), C-X-C motif chemokine receptor 4 (CXCR4, GTX22074), G protein-coupled receptor 85 (GPR85, GTX55244), E-cadherin (GTX100443), GAPDH (GTX100118), N-cadherin (GTX101141), snail1 (GTX122662), vimentin (GTX100619), intercellular adhesion molecule 1 (ICAM1, GTX100450), vascular adhesion molecule 1 (VCAM1, GTX110684), matrix metalloproteinase 2, 9, 12 (MMP2, 9, 12, GTX104577, GTX100458, GTX100704), and anti-rabbit IgG antibody (GTX213110-01), anti-rabbit polyclonal antibody (GTX213110) and anti-mouse monoclonal antibody (GTX300120) were purchased from GeneTex (Irvine, CA, USA). IKKα (SC7218) were purchased from Santa Cruz Biotechnology (Dallas, TX, USA). DharmaFECT1 transfection reagent was purchased from Dharmacon Research (Lafayette, CO, USA) and Lipofectamine 3000 transfection reagent was purchased from Thermo Fisher (Waltham, MA, USA). Novolink polymer detection system for IHC kit was purchased from Leica (Wetzlar, HE, BRD). Recombinant human BRAK (CXCL14) was purchased from PeproTech (Cat. No. 300-50, Rocky Hill, NJ, USA). All other chemicals were purchased from Sigma-Aldrich (St. Louis, MO, USA).

### Cell lines

NSCLC cells (H1299) and human embryonic kidney cells (293T) were purchased from American Type Culture Collection (Manassas, VA, USA). NSCLC cells (A549), normal lung fibroblasts (MRC-5) and human embryo kidney cells (293T) were purchased from the Bioresource Collection and Research Center (BCRC, Hsinchu, Taiwan). H1299 was maintained in RPMI-1640 medium supplemented with 10% fetal bovine serum (FBS), 100 U/ml penicillin, and 100 μg/ml streptomycin. A549 was maintained in F-12 with the same supplement. MRC-5 was maintained in EMEM with the same supplement as mentioned above. 293T was maintained in DMEM with the same supplement as mentioned above. All cells were incubated at 37 °C in a suitable incubator with 5% CO_2_ in an air atmosphere.

### Antibody neutralization

In experiments using antibody neutralization, cells were pre-incubated with ACKR2, CXCR4, GPR85, or IgG antibodies for 1 h prior to subsequent experiments.

### Cell migration assay

Cells (2 × 10^4^ cells/well) were seeded in the upper chamber of Transwell inserts (Cat. No. 3428, 8-μm pore size; Costar, New York, NY, USA). After 24 h, cells were fixed with 4% formaldehyde for 15 min at room temperature and then stained with 0.05% crystal violet for another 45 min. The cells in the upper chamber were removed by swab and the migrated cells were photographed and counted. All experiments were carried out in triplicate wells and were repeated at least three times analyzed by ImageJ 1.52a (National Institutes of Health, Bethesda, MD, USA).

### Wound healing assay

H1299, A549, and MRC-5 (2.5 × 10^4^ cells) were seeded in Culture-Insert (Cat No. 80209, ibidi GmbH, Munich, BRD). After incubation of cells for 24 h, the inserts were removed and the effect of CXCL14 of wound healing was visualized by the microscope at 0 and 24 h. The wound healing area was photographed and counted. All experiments were carried out in triplicate wells and repeated at least three times and analyzed with ImageJ 1.52a.

### Immunohistochemistry

Lung cancer tissue array (LC483a, US Biomax, MD, USA) and animal tissue sections were deparaffinized in xylene, rehydrated in ethanol and washed in deionized water. Novolink polymer detection system was used to block intrinsic peroxidase activity and nonspecific antibody binding sites. The sections were incubated with appropriate primary antibodies (1:200) specific for CXCL14, ACKR2, and N-cadherin. The IHC images were scored taking into account the percentage of positive detection (0-100) by Image J 1.52a and the intensity of staining (0-3+), providing a final score ranging from 0 to 300.

### Western blot

Proteins were resolved using sodium dodecyl sulfate-polyacrylamide gel electrophoresis and transferred to polyvinyl difluoride membranes (Merck Millipore, Burlington, MA, USA). The membranes were probed with primary antibodies (1:1000) at 4 °C overnight. The membranes were subsequently incubated with secondary antibody (1:10,000) at room temperature for 1 h. The blots were developed through enhanced chemiluminescence (RPN2235, AmershamTM cytiva, Washington, D.C., USA) and visualized using chemiluminescence detection system (UVP ChemiDOC-It 815, Analytik Jena US, CA, USA).

### Enzyme-linked immunosorbent assay (ELISA)

The secretion of CXCL14 from cells was evaluated using human CXCL14/BRAK DuoSet ELISA as described by the manufacturer (Cat No. DY866 R&D systems, Minneapolis, MN, USA). Briefly, FBS-free cultured medium was collected from plates after incubation with 5 × 10^5^ cells for 24 h. The samples were observed with a detection antibody and developed by streptavidin-HRP and substrate solution, and the absorbance at 450 nm was determined using a microplate reader (VARIOSKAN LUX, Thermo Fisher, Waltham, MA, USA).

### Cell viability assay

Cells (1 × 10^4^) were seeded in each well in a 48-well plate and changed to a medium containing different concentrations of CXCL14 on day 2. After 24 h, 10 μl of CCK-8 (Cat. No. 96992, Sigma-Aldrich) was added to each well and incubated for 4 h. The microplate was measured at absorbance of 450 nm with the VARIOSKAN LUX microplate reader (Thermo Fisher Scientific, Waltham, MA, USA).

### Immunofluorescence

After treatment, cells were fixed and incubated with appropriate primary antibodies (1:200) specific for E-cadherin, ZO-1, N-cadherin, vimentin, or p65 at 4 °C overnight. After washing, the secondary goat anti-rabbit IgG antibody conjugated with FITC was used for 1 h at room temperature. Cells were then incubated with a DAPI solution for 5 min and examined using a Nikon ECLIPSE Ti microscope (NIS Elements AR 5.02.01).

### Transient transfections

PLC, PKC, c-Src, p65, ACKR2, CXCR4, GPR85 and control siRNAs were purchased from Sigma-Aldrich (St. Louis, MO, USA) and performed with DharmaFECT1 transfection reagent for 24 h according to the manufacturer's instructions. The siRNA sequences used in this study were PLC (5′-GAUGUACCGCCAGGCACUA-3′), PKC (5′-GAGUUUCGGAGCUGAUGAA-3′), c-Src (5′-CAGUUGUAUGCUGUGGUUU-3′), p65, 5′-GGAAUCCAGUGUGUGAAGA3′), and ACKR2 (5′-GCACUCUUUAUACUAUUAA3′), CXCR4: 5'-CACUCCAAGGGCCACCAGA-3', GPR85: 5'-CUUGCAAAGUGAUUGCCUU-3'.

### Reporter gene assay

Cells (2 × 10^5^) were seeded in each well in 12-well plates and transfected with NF-κB promoter reporter plasmid (Promega, Madison, WI, USA). Lipofectamine 3000 transfection reagent was used for 24 h. Cells were lysed using 100 μL reporter lysis buffer (E153A, Promega, Madison, WI, USA). Suspension (50 μL) were placed in wells of an opaque white 96-well microplate and 50 μL of luciferase substrate (Luciferase Assay System E151A, Promega, Madison, WI, USA) was added. For the investigation of signaling pathways, transfected cells were pretreated with PLCβ3, PKCα, c-Src, NF-κB inhibitors and then incubated with CXCL14 for another 24 h. Relative luciferase activity was measured using a microplate luminometer (VICTOR X2, Perkin Elmer, MA, USA).

### Lentivirus transduction and generation of stable cell lines

CXCL14 human-tagged ORF clone lentiviral particle was purchased from ORiGENE (Rockville, MD, USA). Short hairpin (shRNA) targeting the CXCL14 and eGFP-luciferase plasmid was purchased from the National RNAi Core Facility Platform (RNAi Core, Taipei, Taiwan). The target sequence of the CXCL14 shRNA was 5′-GATCCGCTACAGCGACGTGAA-3′. 293T cells were transfected with an empty vector, CXCL14 shRNA or human-tagged ORF clone lentiviral particle CXCL14 for 24 h in a medium containing lentiviral particles. H1299 or A549 cells were incubated with a medium containing lentiviral particles to generate stable CXCL14 shRNA-luciferase-GFP cells expressing CXCL14 shRNA or overexpressing CXCL14-luciferase-GFP cells.

### Quantitative real-time polymerase chain reaction (qPCR)

RNA was extracted using an easy-BLUE total RNA extraction kit (iNtRON Biotechnology, Seongnam-si, Gyeonggi-do, KOR). First-stand cDNA was obtained using a qPCRBIO cDNA Synthesis Kit (Cat. No. PB30.11-10, PCR biosystems, London, England, UK). Human *CXCL14*, *CDH1*, *CDH2*, *VIM*, *SNAI1*, and *GAPDH mRNA expression* was examined by qPCR. The KAPA SYBR FAST qPCR Master Mix (2×) kit (Cat. No. KK4600, Sigma-Aldrich) on a CFX Connect real-time PCR detection system (BioRad, Hercules, CA, USA) was used according to the manufacturer's instructions. All primers were purchased from Sigma‐Aldrich. *GAPDH* was used as an internal control. Gene expression levels were performed using the 2^-ΔΔCt^ method. The primer sequences used for qPCR were *CXCL14* (forward: 5′-AAAACCTCAGAAGGGAAAAC-3′, reverse: 5′-ATTTGTGTCTTATGCCTGTG-3′), *CDH1* (E-cadherin, forward: 5′-CCGAGAGCTACACGTTC-3′, reverse: 5′-TCTTCAAAATTCACTCTGCC-3′), *CDH2* (N-cadherin, forward: 5′-ACATATGTGATGACCGTAAC-3′, reverse: 5′-TTTTTCTCGATCAAGTCCAG-3′), *VIM* (vimentin, forward: 5′-GGAAACTAATCTGGATTCACTC-3′, reverse: 5′-CATCTCTAGTTTCAACCGTC-3′), *SNAI1* (Snail1, forward: 5′-CTCTAATCCAGAGTTTACCTTC-3′, reverse:5′-GACAGAGTCCCAGATGAG-3′), and *GAPDH* (forward: 5′-ACAGTTGCCATGTAGACC-3′, reverse: 5′-TTGAGCACAGGGTACTTA-3′).

### *In vivo* orthotopic lung cancer model

An orthotopic lung cancer model was modified as described in previous studies 20, 21. Four-week-old male BALB/C nude mice were anesthetized with 2% isoflurane. A cell suspension of H1299 (1 × 10^6^ cells) with a total volume of 50 μL (RPMI medium: Matrigel = 1:1) were injected directly into the left lung. Three weeks after injection, the tumor was examined with an IVIS Luminar II *in vivo* imaging system (PerkinElmer, Waltham, MA, USA). All the right and left lungs were collected and embedded in paraffin. All animal experiments were conducted according to the protocols approved by the Institutional Ethics Committee of Shin Kung Wu Ho Su Memorial Hospital (IACUC Approval No. 111MOST006).

### Statistical analysis

Data are presented as mean ± standard deviation (SD). Two groups of data were compared using Student's t test. More than two groups of data were compared using one‐way analysis of variance using GraphPad Prism 8.0. Statistical significance is represented in figures by: *, *p* value < 0.05; **, *p* value < 0.01; ***, *p* value < 0.001; ****, *p* value < 0.0001.

## Results

### CXCL14 is highly expressed and correlates with the clinical stage of lung cancer

**Figure 1:**
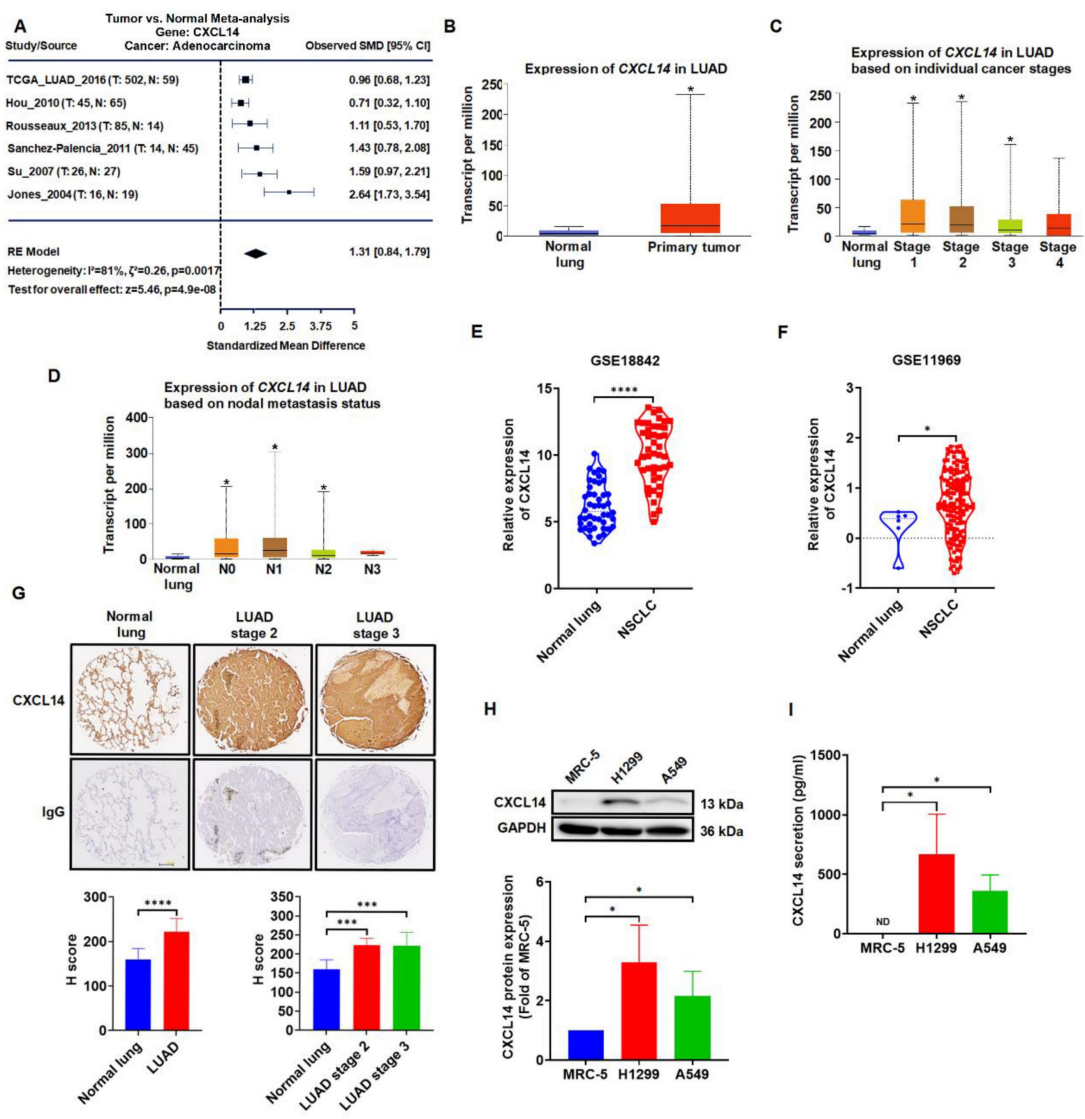
** CXCL14 is overexpressed in lung cancer. (A)** Difference in *CXCL14* expression between normal and lung adenocarcinoma (LUAD) tissues was analyzed by meta-analysis using data downloaded from Lung Cancer Explorer (LCE). (B-D) The gene expression of *CXCL14* in normal lung and LUAD obtained from The Cancer Genome Atlas (TCGA). (E, F) *CXCL14* mRNA expression in NSCLC and normal lung tissues in GSE18842 and GSE11969 from the Gene Expression Omnibus (GEO) database. **(G)** Expression of CXCL14 in normal and tumor lung tissues stained with immunohistochemistry (IHC) and different stages was analyzed using the H score (n = 8). Scale bar = 200 μm. **(H)** The expression of the CXCL14 protein in MRC-5, H1299, and A549 cells was examined using Western blot (n = 4). **(I)** CXCL14 secretion was examined in MRC-5, H1299, and A549 cells using an enzyme-linked immunosorbent assay (ELISA; n = 4). Normal tissues and MRC-5 cells were used as controls.

Meta-analysis data sets were analyzed to explore the expression of CXCL14 in normal lung tissue and lung tumor. We first examined the expression profile of CXCL14 in lung cancer tissues by using online lung cancer‐specific database‐the Lung Cancer Explorer (LCE). Meta-analysis showed that CXCL14 was upregulated in lung cancer tissues containing adenocarcinoma (LUAD) (Figure 1A). TCGA gene analysis indicated that CXCL14 was highly associated with the clinical stage of the disease and metastatic lung cancer of the nodule (Figure 1B-D). Furthermore, analysis of the Gene Expression Omnibus (GEO) data set (GSE18842 and GSE11969) indicated that the expression levels of CXCL14 in NSCLC patients were significantly higher than in normal samples (Figure 1E-F). Interestingly, the IHC scores of lung cancer tissue array were similar to Meta-analysis and GEO datasets indicated that CXCL14 expression was significantly upregulated in the lung cancer tissues (Figure 1G). These observations were confirmed by ELISA and Western blot analysis of CXCL14 levels in normal and cancer cell lines. The level of CXCL14 expression was higher in the cancer cells (Figure 1H-I). Collectively, the expression level of CXCL14 was overexpressed in lung cancer and patients with metastases. These results suggest that CXCL14 plays a pivotal role in lung cancer metastasis.

### CXCL14 promotes the migration of lung cancer cells via EMT

**Figure 2:**
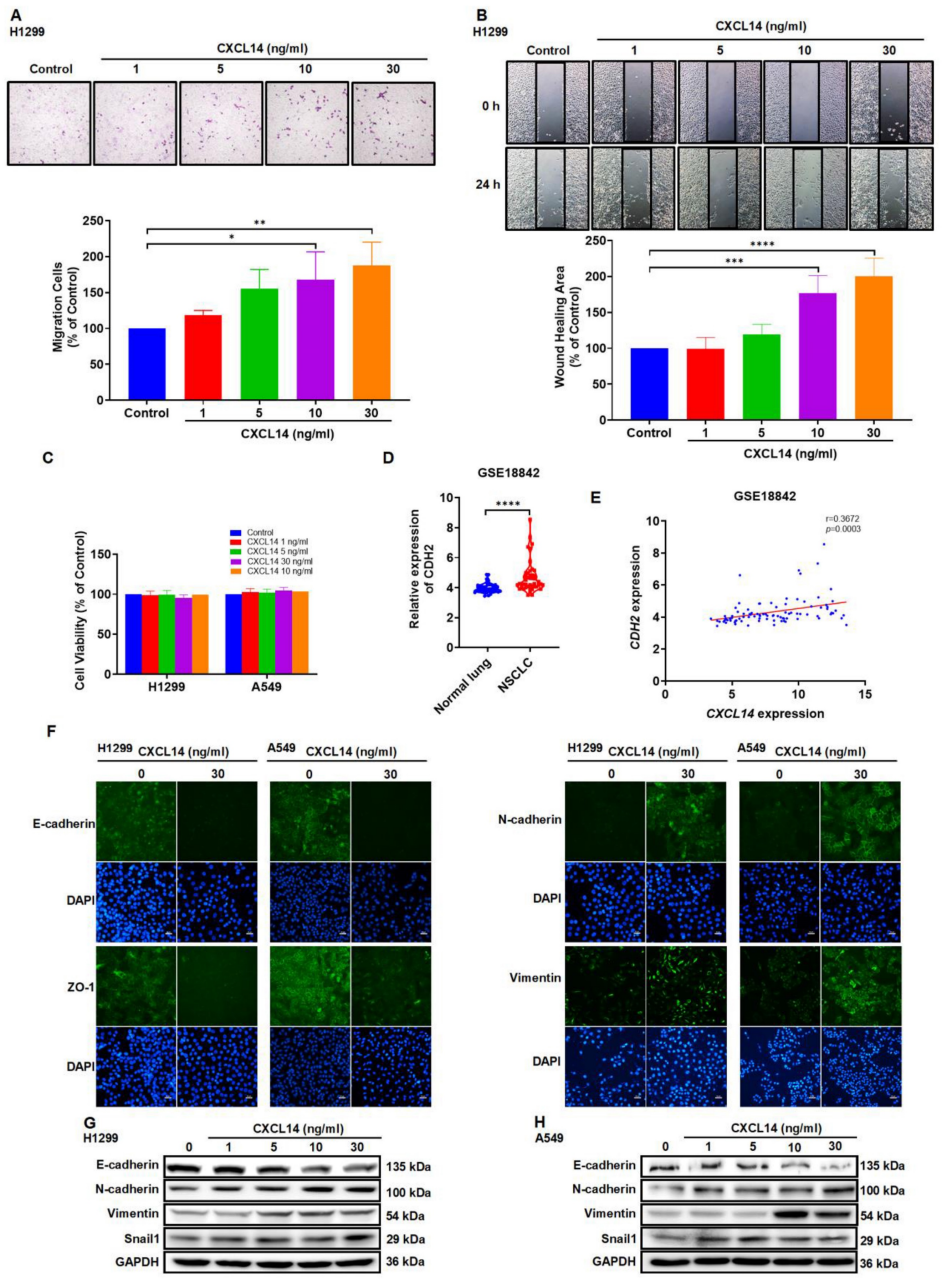
** CXCL14 promotes migration and regulates the epithelial mesenchymal transition in lung cancer cells. (A, B)** H1299 cells were treated with CXCL14 (1-30 ng/ml) for 24 h, cell migration was measured with migration and wound healing assays (n = 4). **(C)** H1299 and A549 cells were treated with CXCL14 (1-30 ng/ml) for 24 h, cell viability was evaluated using a CCK-8 assay (n = 4). **(D)** Expression of *CDH2* mRNA in NSCLC and normal lung tissue in GSE18842 from the GEO database. **(E)** Correlation analysis of *CXCL14* and *CDH2* in GSE18842. **(F)** After treatment with CXCL14 (30 ng/ml) for 24 h, the expression levels of E-cadherin, ZO-1, N-cadherin and vimentin were determined in H1299 and A549 cells on immunofluorescence (IF)-stained slides. Scale bar = 20 μm. **(G, H)** After incubation with CXCL14 (1-30 ng/ml) for 24 h, the expression of epithelial mesenchymal transition (EMT) markers in the H1299 and A549 cells were examined by Western blot (n = 4). Untreated cells were used as controls.

On the basis of the above findings, we examined the function of CXCL14 in lung cancer cells. CXCL14-dependent stimulation of cells promoted cell motility but did not affect lung cell proliferation (Figure 2A-C and S1A-B). EMT promotes cell migration and contributes to cancer cell metastasis 22. Our investigation of GSE18842 NSCLC tissue samples from the GEO data set identified significantly higher N-cadherin expressions in NSCLC and we observed a positive correlation between CXCL14 and N-cadherin levels (Figure 2D-E). Next, to investigate the relationship between CXCL14 and EMT, the expression of epithelial cell markers, E-cadherin, ZO-1; mesenchymal cell markers, N-cadherin, and vimentin were examined in H1299 and A549 cells. Stimulation of A549 and H1299 with CXCL14 promoted N-cadherin, vimentin, and snail1 and decreased the expression of E-cadherin and ZO-1 according to immunofluorescence and Western blot assays (Figure 2F-H). MMPs enhance cancer cell migration and promote tumor development 23, 24. Several adhesion molecules, such as vascular cellular adhesion molecule (VCAM)-1 and intercellular adhesion molecule (ICAM)-1, exhibit increased expression in metastatic cancer 25, 26. Therefore, we investigated the expression of MMPs and CAMs in lung cancer cells. Treated A549 and H1299 cells with CXCL14 did not affect the expression of ICAM-1, VCAM-1 and MMPs (Figure S1C-D). These results revealed that EMT was positively associated with the migration of CXCL14-promoted lung cancer cells.

### ACKR2 is essential for CXCL14-induced cell migration in lung cancer cells

**Figure 3:**
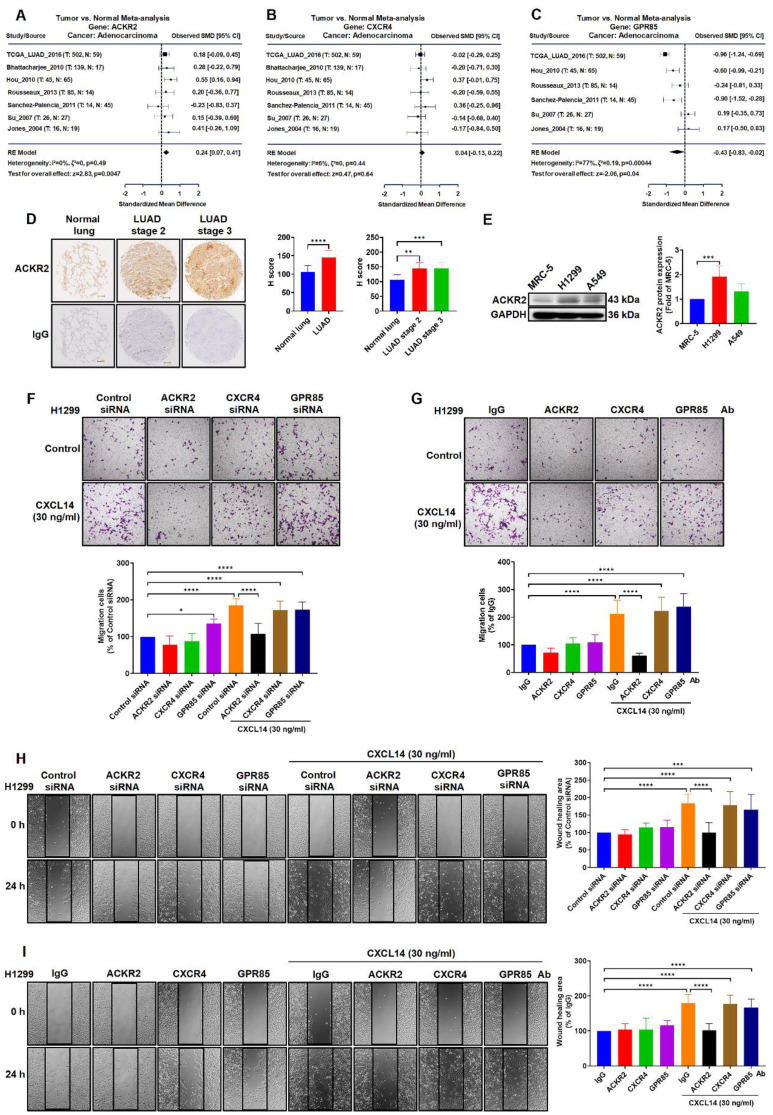
** ACKR2 is overexpressed and regulates migration in lung cancer. (A-C)** Differences in *ACKR2*, *CXCR4*, and *GPR85* expression between normal and lung adenocarcinoma (LUAD) tissues were determined through meta-analysis. **(D)** The expression levels of ACKR2 in immunohistochemistry (IHC)-stained normal and different stage of tumor lung tissues were analyzed using the H score (n = 8). Scale bar = 200 μm. **(E)** The expression of the ACKR2 protein in MRC-5, H1299 and A549 cells was examined by Western blot. Data are shown as fold of MRC-5 (n = 4). **(F, H)** H1299 cells were transfected with control siRNA or specific ACKR2, CXCR4, and GPR85 siRNA for 24 h and incubated with CXCL14 (30 ng/ml) for another 24 h (n = 4). Cell migration was assessed through migration and wound healing assays. **(G, I)** H1299 cells were incubated with IgG or ACKR2, CXCR4, and GPR85 neutralized antibodies (1 μg/ml) for 1 h and incubated with CXCL14 (30 ng/ml) for another 24 h (n = 4). Cell migration was assessed through migration and wound healing assays. Normal tissues, MRC-5 cells, cells transfected with control siRNA, and incubated with IgG were used as controls.

Several studies have reported that CXCL14-induced molecular signaling and cellular responses depend on ACKR2, CXCR4, and GPR85 in many different tumor cells 19, 27, 28. Therefore, we investigated the role of ACKR2, CXCR4, and GPR85 in lung cancer. A meta‐analysis in all lung cancer tissues containing adenocarcinoma (LUAD) found that the expression of ACKR2 but not the other candidate receptors, CXCR4 and GPR85, was up-regulated in lung cancer studies (Figure 3A-C). Results of IHC staining and western blotting for levels of ACKR2 in lung cancer were similar to the analysis of TCGA database samples, showing higher levels of ACKR2 expression in lung cancer tissues and lung cancer cells (Figure 3D-E). Next, lung cancer cells transfected with the ACKR2 siRNA or incubated with ACKR2 neutralized antibody markedly abolished CXCL14‐induced cell migration and cell movement, but not CXCR4, and GPR85 (Figure 3F-I and S2A-D). These results showed that ACKR2 receptor plays a crucial role for CXCL14 to induce migration in lung cancer cells.

### CXCL14 induces cancer cell migration through an ACKR2-dependent signaling pathway

**Figure 4:**
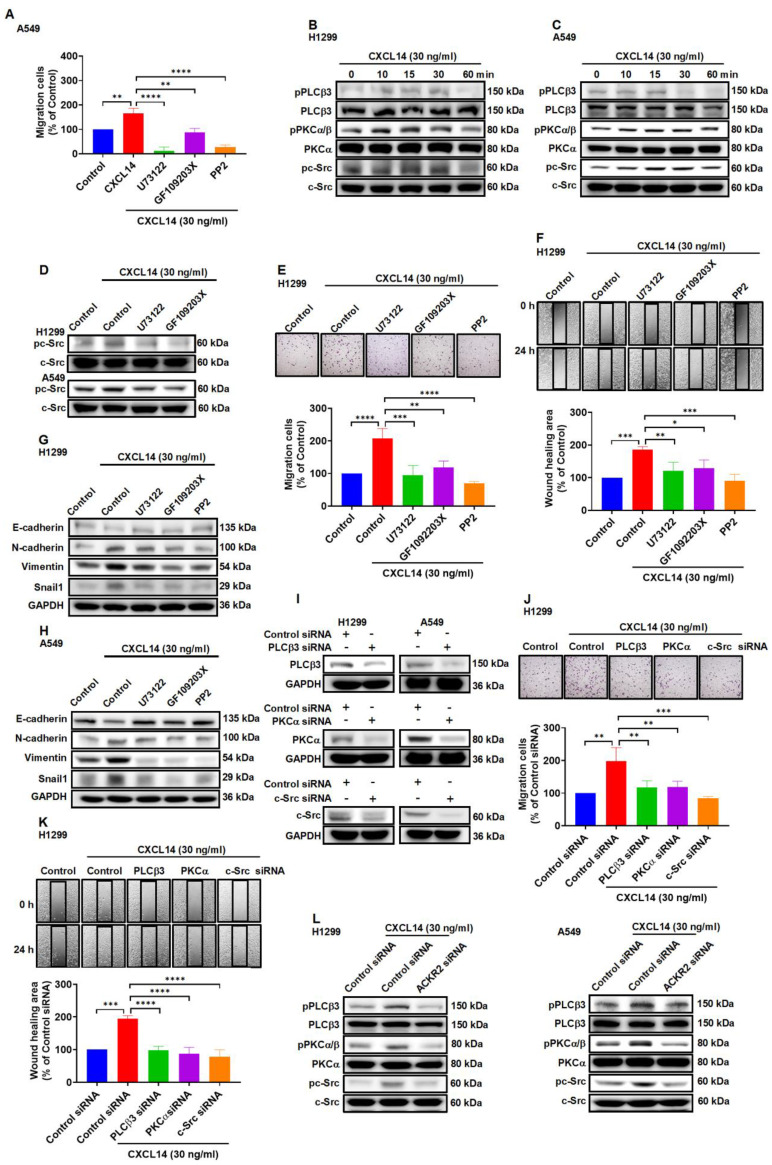
** CXCL14 promotes cell migration and EMT marker expression via ACKR2 and PLCβ3, PKCα and c-Src in lung cancer cells. (A)** A549 cells were pretreated with U73122 (3 μM), GF109203X (1 μM) or PP2 (1 μM) for 1 h and then treated with CXCL14 (30 ng/ml) for 24 h. Cell migration was measured using a migration assay (n = 4). **(B-C)** H1299 and A549 cells were treated with CXCL14 (30 ng/ml) and the phosphorylation of PLCβ3, PKCα, and c-Src was examined using Western blot (n = 4). **(D)** H1299 and A549 cells were pretreated with U73122 or GF109203X for 1 h and then treated with CXCL14 (30 ng/ml) for 15 min. Phosphorylation of c-Src was measured using Western blot (n = 4). **(E, F)** H1299 cells were pretreated with U73122, GF109203X or PP2 for 1 h and then incubated with CXCL14 (30 ng/ml) for 24 h. Cell migration was measured with migration and wound healing assays (n = 4). **(G, H)** H1299 and A549 cells were pretreated with U73122, GF109203X or PP2 for 1 h and then treated with CXCL14 (30 ng/ml) for 24 h. The expression of EMT markers was examined using Western blot (n = 4). **(I)** H1299 and A549 cells were transfected with control siRNA or siRNA specific for PLCβ3, PKCα, or c-Src, and transfection efficiency was measured by Western blot (n = 4). **(J, K)** H1299 cells were transfected with control siRNA or siRNA specific for PLCβ3, PKCα, or c-Src and then incubated with CXCL14 (30 ng/ml) for 24 h. Cell migration was measured using migration and wound healing assays (n = 4). **(L)** H1299 and A549 cells were transfected with control siRNA or siRNA specific for ACKR2 siRNA and incubated with CXCL14 (30 ng/ml) for 15 min. The phosphorylation of PLCβ3, PKCα, and c-Src was examined using Western blot (n = 4). Untreated cells and cells transfected with control siRNA were used as controls.

Previous studies revealed that activation of the phospholipase C (PLC), protein kinase C (PKC) and c-Src pathways was involved in metastatic development to promote tumor progression 29-31. To examine the effect of PLC, PKC, and c-Src in CXCL14-promoted migration. A549 cells were treated with PLCβ3 inhibitor (U73122), protein kinase Cα (PKCα) inhibitor (GF109203X), and proto-oncogene inhibitor c-Src (c-Src) (PP2), which abolished the migration of cells induced by CXCL14 (Figure 4A). Western blot analysis demonstrated that CXCL14 time-dependently promoted the phosphorylation of PLCβ3, PKCα, and c-Src in H1299 and A549 cells (Figure 4B-C). Pretreatment with U73122 and GF109203X suppressed c-Src phosphorylation activated by CXCL14 confirmed c-Src-dependent PLCβ3 and PKCα activation mediates CXCL14-induced cell migration in lung cancer cells (Figure 4D). The PLCβ3, PKCα, and c-Src inhibitors and siRNAs abolished CXCL14-induced cell motility and EMT in H1299 and A549 cells (Figure 4E-K and S3A-D). Moreover, transfection of ACKR2 siRNA prevented CXCL14-activated PLCβ3, PKCα, and c-Src phosphorylation (Figure 4L), indicating that CXCL14 promoted lung cancer cell migration and EMT through activation of ACKR2/PLCβ3/PKCα/c-Src signaling pathway.

### CXCL14 initiates EMT and cell migration by activating nuclear factor-κB

**Figure 5:**
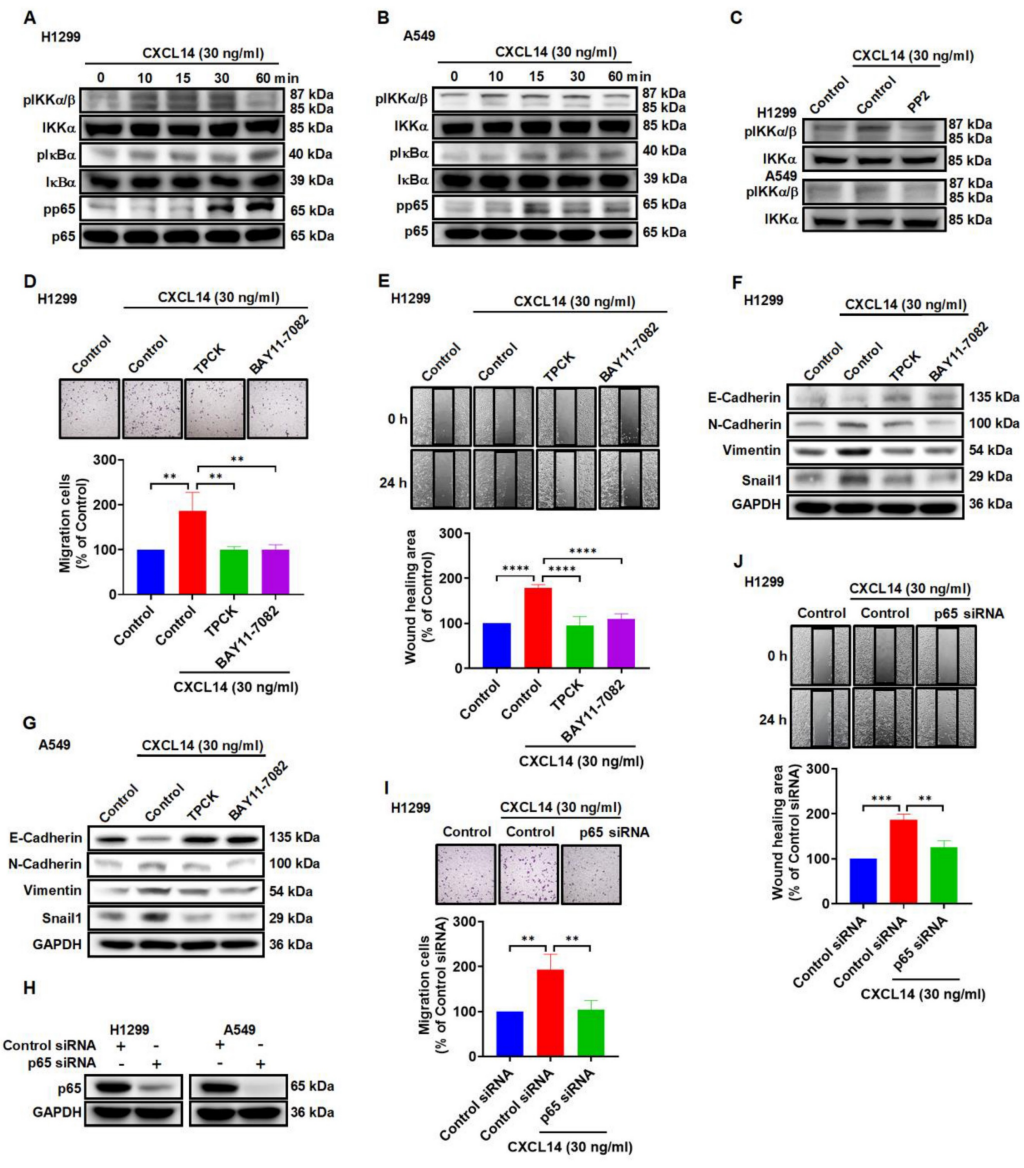
** CXCL14 promotes cell migration and EMT marker expression through the IKKα, IκBα, and p65 in lung cancer cells. (A, B)** H1299 and A549 cells were treated with CXCL14 (30 ng/ml) to detect the phosphorylation of IKKα, IκBα, and p65 by using Western blot (n = 4). **(C)** H1299 and A549 cells were pretreated with PP2 for 1 h and treated with CXCL14 (30 ng/ml) for 15 min. The phosphorylation of IKKα was measured using Western blot (n = 4). **(D, E)** H1299 cells were pretreated with TPCK (1 μM) or BAY11-7082 (0.6 μM) for 1 h and incubated with CXCL14 (30 ng/ml) for 24 h. Cell migration was measured through migration and wound healing assays (n = 4). **(F, G)** H1299 and A549 cells were pretreated with TPCK or BAY11-7082 for 1 h and incubated with CXCL14 (0 or 30 ng/ml) for 24 h. The expression of EMT markers was examined by Western blot (n = 4). **(H)** H1299 and A549 cells were transfected with control siRNA or siRNA specific for p65 siRNA, and transfection efficiency was measured using Western blot (n = 4). **(I, J)** H1299 cells were transfected with siRNA specific for p65 and treated with CXCL14 (30 ng/ml) for 24 h. Cell migration was measured through migration and wound healing assays (n = 4). Untreated cells and cells transfected with control siRNA were used as controls.

Nuclear factor-κB (NF-κB) is considered to be involved in EMT and metastasis in the lung and other types of cancer 32-34. In this study, stimulation of lung cancer cells with CXCL14 promoted IKKα, IκBα, and p65 phosphorylation, which was antagonized by the c-Src inhibitor (Figure 5A-C). To verify direct CXCL14 activation of NF-κB mediating EMT and migration, we pretreated lung cancer cells with the inhibitors of IκB kinase α (IKKα) and NF-κB inhibitor α (IκBα) or p65 siRNA to evaluate CXCL14-promoted cell migration and EMT. We found that CXCL14-induced cell migration and EMT were suppressed by IKKα and IκBα inhibitors and p65 siRNA (Figure 5D-J and S4A-D). The results illustrated that NF-κB activation is necessary for CXCL14-promoted migration and EMT in lung cancer cells.

### CXCL14 promotes NF-κB transcriptional activity via the PLCβ3, PKCα, and c-Src pathways

**Figure 6:**
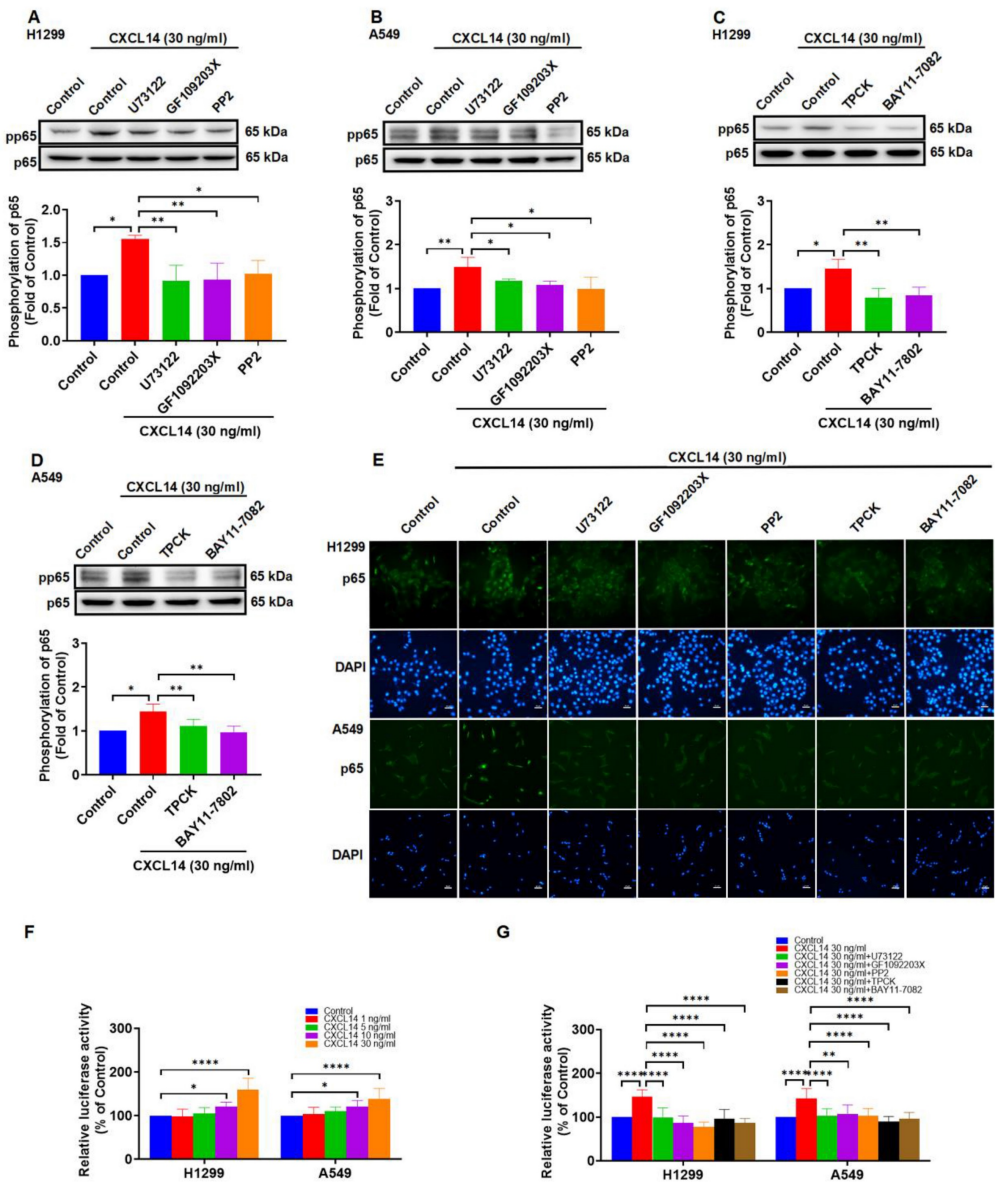
** CXCL14 promotes NF-κB transcriptional activation via PLCβ3, PKCα, c-Src, IKKα, and IκBα in lung cancer cells. (A, B)** H1299 and A549 cells were pretreated with U73122, GF109203X, or PP2 for 1 h and incubated with CXCL14 (30 ng/ml) for 1 h. The phosphorylation of p65 was examined using Western blot (n = 4). **(C, D)** H1299 and A549 cells were pretreated with TPCK or BAY11-7082 for 1 h and incubated with CXCL14 (30 ng/ml) for 1 h. The phosphorylation of p65 was examined using Western blot (n = 4). **(E)** H1299 and A549 cells were pretreated with U73122, GF109203X, PP2, TPCK or BAY11-7082 for 1 h and incubated with CXCL14 (30 ng/ml) for 3 h. Cells were stained with anti-p65 antibody and analyzed under an immunofluorescence microscope. The nuclei were counterstained with DAPI. Representative microscopic images are shown. Scale bar = 20 μm. **(F)** H1299 and A549 cells were transfected with an NF-κB promoter reporter plasmid for 24 h, treated with CXCL14 (1-30 ng/ml) for another 24 h, and evaluated in terms of luciferase activity (n = 4). **(G)** H1299 and A549 cells were transfected with an NF-κB promoter reporter plasmid for 24 h and pretreated with U73122, GF109203X, PP2, TPCK or BAY11-7082 for 1 h. Cells were incubated with CXCL14 (30 ng/ml) for another 24 h and assessed in terms of luciferase activity (n = 4). Untreated cells were used as controls.

To explore the mechanism of CXCL14 in NF-κB activation, treating lung cancer cells with PLCβ3, PKCα, c-Src inhibitors reversed CXCL14-mediated activation of p65 (Figure 6A-B). Besides, IKKα and IκBα inhibitors showed the dependency of specific signaling on CXCL14-induced activation of p65 (Figure 6C-D). As shown in Figure 6E-G, CXCL14 stimulation of cells increased p65 translocation into the nucleus and NF-κB luciferase activity. These effects were suppressed by PLCβ3, PKCα, c-Src, IKKα, and IκBα inhibitors (Figure 6E-G). These results demonstrated that CXCL14 promoted NF-κB transcriptional activity through the PLCβ3, PKCα, and c-Src pathways.

### CXCL14 induces tumor metastases *in vivo* orthotopic lung cancer model

**Figure 7:**
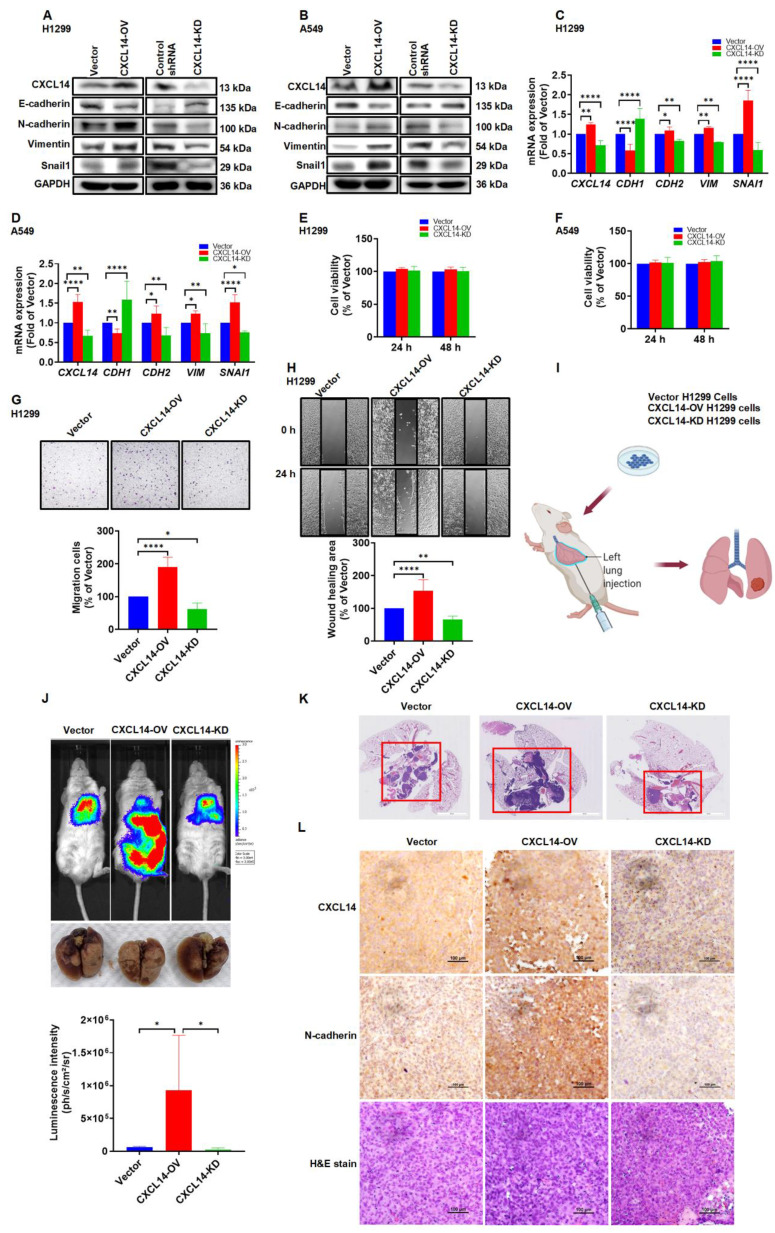
** CXCL14 promotes tumor metastasis in the orthotopic model. (A, B)** The transfection effects of CXCL14 (OV or KD) in H1299 and A549 cells were examined by Western blot (n = 4). **(C, D)** The effects of transfection in H1299 and A549 cells were measured using a quantitative real-time polymerase chain reaction (n = 4). **(E, F)** Cell proliferation of CXCL14-OV and CXCL14-KD cells was examined using a CCK-8 assay (n = 4). **(G, H)** Cell migration of CXCL14-OV and CXCL14-KD H1299 cells was measured with migration and wound healing assays (n = 4). **(I)** Schematic illustration of the orthotopic model. **(J)** Representative images of lung metastases of H1299, CXCL14-OV and CXCL14-KD H1299 vector cells were measured with an *in vivo* imaging system. The bottom panel showed *ex vivo* lung tumor images and luminescence intensity of each tumor (n = 4). **(K)** Representative H&E staining of metastatic lung tumor nodules in each group (n = 4). Scale bar = 4 mm. **(L)** Representative IHC and H&E staining of orthotopic lung tumor in each group (n = 4). Scale bar = 100 μm.

To further investigate the role of CXCL14 in lung cancer metastasis, we generated cells with stable overexpressed CXCL14 (CXCL14-OV) and cells with knocked down CXCL14 (CXCL14-KD). CXCL14 overexpression cells promoted high levels of both the CXCL14 and EMT protein, which significantly increased the migration ability of H1299 and A549 cells (Figure 7A-D, G-H and S5A-B) but not cellular proliferation (Figure 7E-F). H1299 (Vector), CXCL14-OV, and CXCL14-KD were orthotopically implanted in the left lung and cancer metastasis was analyzed using an *in vivo* imaging system (IVIS). IVIS data revealed that CXCL14 overexpression significantly exhibited a higher bioluminescence intensity (BLI), and the BLI was reduced in the CXCL14-KO group compared to that of the vector group (Figure 7J). The H&E stain images showed that CXCL14 overexpression increased the tumor distribution area in the left and right lung lobes, and the area in the CXCL14-KD group was lower than in the vector group (Figure 7K). Interestingly, a positive correlation between CXCL14 and N-cadherin protein expression was observed in mouse lung tissue (Fig. 7L). Our data indicated that endogenous CXCL14 increased EMT-dependent cancer metastasis of lung cancer in mice.

## Discussion

Lung cancer metastasis is the main cause of the treatment dilemma 4, 5. Identifying crucial molecules that affect cancer cell metastasis is vital in therapeutic strategies. CXCL14 is overexpressed in lung cancer in the TCGA database and the GEO dataset (Figure 1A-B and E-F). However, the function of CXCL14 in lung cancer progression is still unclear. CXCL14 was found to be associated with lymph node metastasis in papillary thyroid carcinoma and to promote proliferation, migration, and invasiveness in colorectal carcinoma cells 35, 36. Additionally, CAF overexpresses and secretes CXCL14 in breast and prostate cancer and promotes tumor growth and invasion compared to normal stromal cells 37-39. Moreover, CXCL14 is associated with a poor prognosis for patients with cervical and endometrial cancer 9, 40. The most interesting observation emerging from our data is that CXCL14 promotes tumor metastasis through ACKR2 in NSCLC cells. CXCL14 is associated with tumor stages and nodal metastasis. These findings support that CXCL14 is an appropriate prognostic and therapeutic marker target.

According to Bal *et al.*, ACKRs are involved in the growth, migration, and invasion in tumor cells 41 and bind to cysteine-cysteine-type and cysteine-X-cysteine-type chemokines. Furthermore, ACKR2 is a receptor responsible for CXCL14-induced EMT and metastasis in breast cancer 19. Additionally, ACKR2 deficiency affects melanoma cell metastasis to the lung regions in a mouse model 42. Previous studies have shown that the high expression of CXCL14/CXCL12 and cell surface receptor CXCR4 is highly correlated with tumor metastasis in endometrioid carcinoma 27. In addition, the activation of cancer-associated fibroblasts through the CXCL14/GPR85 pathway has been shown to leads to EMT and progression in breast cancer 28. In this study, we explored the expression of three candidate receptors of CXCL14 and their role on migration in NSCLC cells. The meta‐analysis data in LUAD found that ACKR2 is overexpressed but not CXCR4 and GPR85. The elimination of ACKR2 decreases the migration of CXCL14-induced cancer cells. This did not markedly inhibit CXCL14-induced cell migration in the group using CXCR4 and GPR85-specific siRNA and neutralized antibodies. The most important aspect of the data was that ACKR2 is an essential receptor for CXCL14-promoted lung cancer metastasis.

Previous studies have shown that ACKRs bind to chemokines and activate the β-arrestin pathway rather than coupling with G proteins to scavenge chemokines and regulate cell homeostasis 43. However, ACKRs were found to regulate kinase phosphorylation and cancer progression in different cancer cells 44-47. In addition, dysregulation of cellular kinases, including, phospholipase, phosphokinase, and c-Src, is involved in proliferation, metastasis, and EMT in cancer progression 48-50. In addition, the transcription factor NF-κB is involved in tumor development and metastasis in lung cancer and other types of cancer 34, 51, 52. The present results revealed that ACKR2 plays a critical role in the progression of CXCL14-induced lung cancer. The elimination of ACKR2 abolished the CXCL14-promoted phosphorylation of PLCβ3, PKCα, and c-Src and lung cancer cells. Additionally, CXCL14 promoted the transcriptional activity of NF-κB, which is related to tumor migration and EMT of lung cancer cells. Furthermore, the inhibition of PLCβ3, PKCα, and c-Src phosphorylation reversed CXCL14-induced transcriptional activity of NF-κB in lung cancer cells. A somewhat remarkable relationship was that CXCL14/ACKR2 promote EMT and cell migration by triggering the activation of PLCβ3, PKCα, and c-Src signaling and transcription factor p65.

Previous studies have shown that mitogen-activated protein kinase (MAPK), focal adhesion kinase (FAK), and Akt signaling pathways have been involved in cell migration and progression in lung cancer 53-55. In this study, we also investigated the role of MAPK, FAK, and Akt in CXCL14-induced cell migration in A549 cells. Our findings showed that the administration of p38 inhibitor (SB203580), JNK inhibitor (SP600125) and FAK inhibitor (FAKi) reduced the migration of CXCL14-induced cells, but not MEK (PD98059 and U0126) (supplementary Figure S3E). These results suggest that further investigation is needed to fully understand the mechanism by which CXCL14 interacts with these signaling pathways and will be the focus of our future work.

## Conclusion

**Figure 8:**
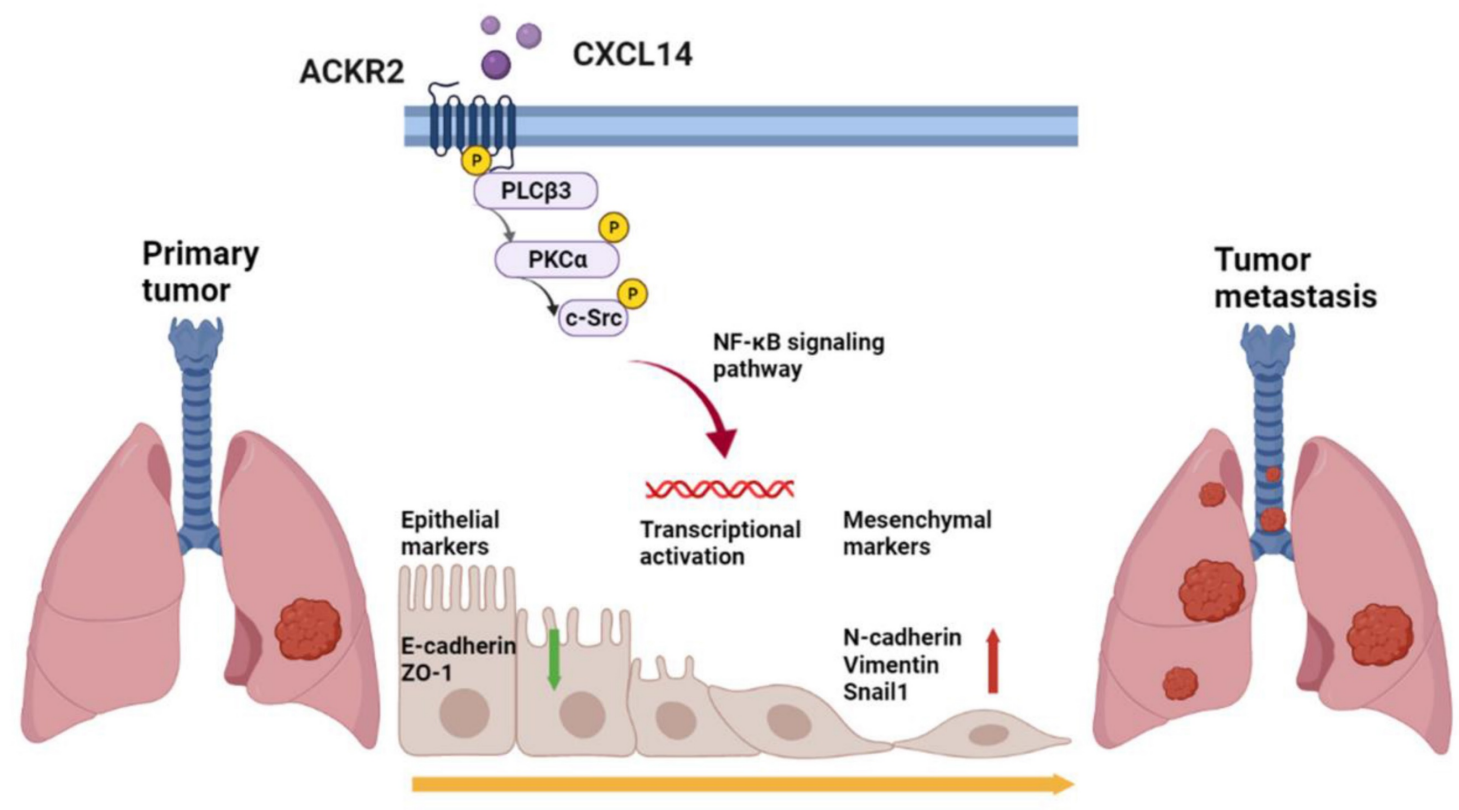
** Schematic mechanism of CXCL14 promotes tumor metastasis in lung cancer.** The ligand CXCL14 stimulates the atypical chemokine receptor 2 (ACKR2) to activate PLCβ3, PKCα, and c-Src. The phosphorylation of phospholipase Cβ3 (PLCβ3), protein kinase Cα (PKCα), and proto-oncogene c-Src (c-Src) signaling pathways activates IκB kinase α (IKKα) and NF-κB inhibitor α (IκBα) to release nuclear factor-κB (NF-κB). The nuclear translocation of NF-κB promotes the expression of EMT protein and cell migration that leads to tumor metastasis.

The results revealed that chemokine CXCL14 promotes EMT and migration through ACKR2 in lung cancer for the first time. These results broaden our understanding of the role and molecular mechanism of CXCL14/ACKR2 interdependence in promoting lung cancer migration *in vitro* and *in vivo* (Figure 8). These findings may provide important information on novel strategies for the clinical diagnosis and treatment of lung cancer metastases.

## Supplementary Material

Supplementary figures.
