# Supplementary material for: CXCL14 promotes metastasis of non-small cell lung cancer through ACKR2-depended signaling pathway
Source: Int J Biol Sci. 2023 Feb 27;19(5):1455–70. doi: 10.7150/ijbs.79438 (PMC10086750; doi:10.7150/ijbs.79438)
Supplement: Supplementary file 1 — Supplementary figures. [file ijbsv19p1455s1.pdf]

Supplementary figures and legends

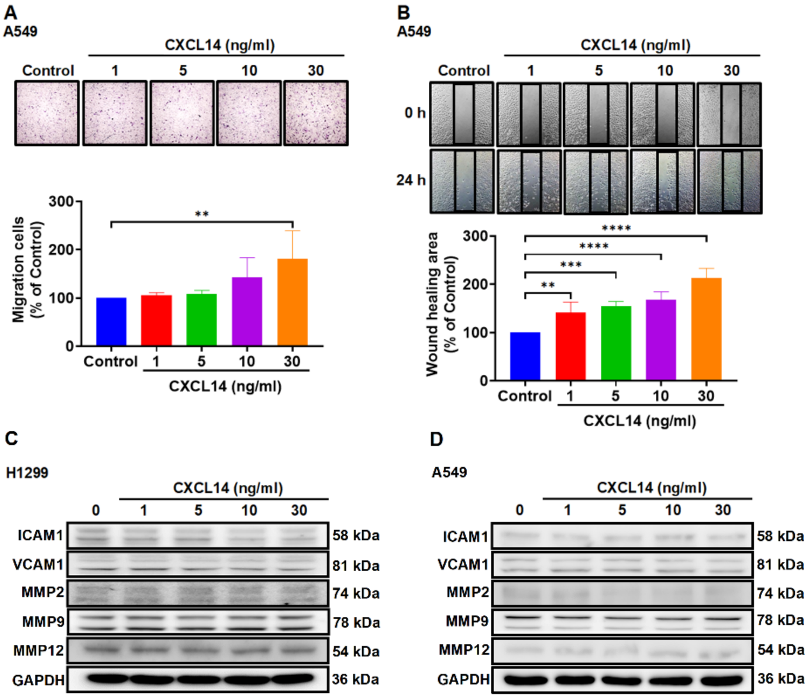

Supplementary Figure S1. CXCL14 promotes migration in lung cancer cells. (A, B) A549 cells were treated with CXCL14 (1–30 ng/ml) for 24 h, cell migration was measured with migration and wound healing assays (n = 4). (C, D) H1299 and A549 cells were treated with CXCL14 (1–30 ng/ml) for 24 h, the expression of adhesion molecules and matrix metalloproteinases (MMPs) was examined by Western blot (n = 4). Untreated cells were used as controls.

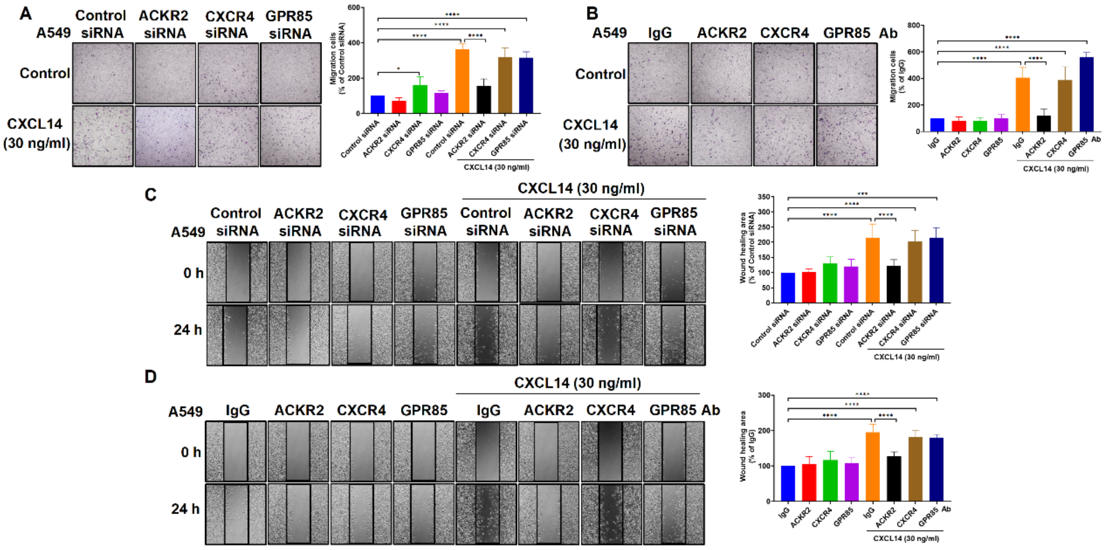

Supplementary Figure S2. ACKR2 regulates migration in lung cancer. (A, C) A549 cells were transfected with nonspecific siRNA or specific ACKR2, CXCR4, GPR85 siRNA for 24 h and incubated with CXCL14 (30 ng/ml) for another 24 h (n = 4). Cell migration was assessed by migration and wound healing assays. (B, D) A549 cells were incubated with ACKR2, CXCR4, GPR85 neutralized antibodies (1 μg/ml) for 1 h and then followed treatment with CXCL14 (30 ng/ml) for another 24 h (n = 4). Cell migration was assessed by migration and wound healing assays. Cells transfected with control siRNA and incubated with IgG were used as controls.

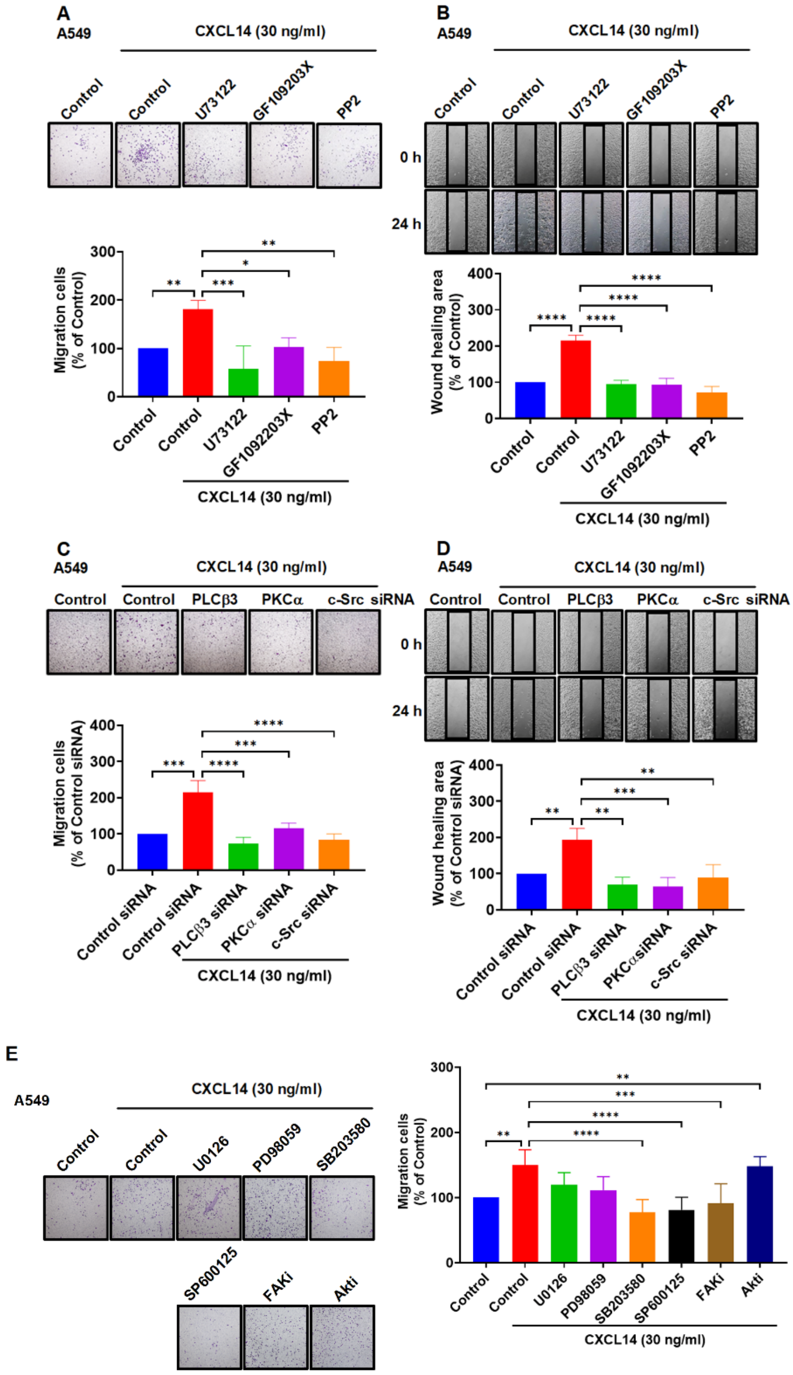

Supplementary Figure S3. CXCL14 promotes cell migration via phosphorylation of PLCβ3, PKCα and c-Src in lung cancer cells. (A, B) A549 cells were pretreated with U73122, GF109203X, or PP2 for 1 h and then incubated with CXCL14 (30 ng/ml) for 24 h. Cell migration was measured with migration and wound healing assays (n = 4). (C, D) A549 cells were transfected with control siRNA or siRNA specific for PLCβ3, PKCα, or c-Src and then incubated with CXCL14 (30 ng/ml) for 24 h. (E) A549 cells were pretreated with U0126, PD98059, SB203580, SP600125, FAKi and Akti for 1 h and then incubated with CXCL14 (30 ng/ml) for 24 h. Cell migration was measured

using migration and wound healing assays (n = 4). Untreated cells and cells transfected with control siRNA were used as controls.

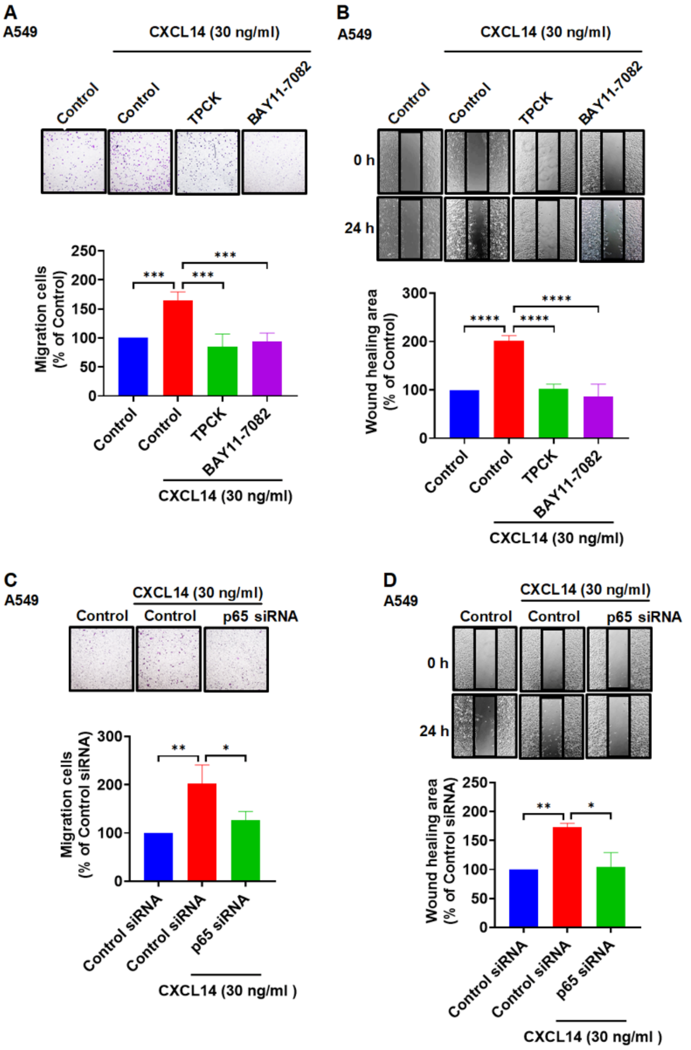

Supplementary Figure S4. CXCL14 promotes cell migration through the phosphorylation of IKKα, IκBα, and p65 in lung cancer cells. (A, B) A549 cells were pretreated with TPCK (1 μM) or BAY11-7082 (0.6 μM) for 1 h and incubated with CXCL14 (30 ng/ml) for 24 h. Cell migration was measured through migration and wound healing assays (n = 4). (C, D) A549 cells were transfected with control siRNA or siRNA specific for p65 and treated with CXCL14 (30 ng/ml) for 24 h. Cell migration was measured through migration and wound healing assays (n = 4). Untreated cells and cells transfected with control siRNA were used as controls.

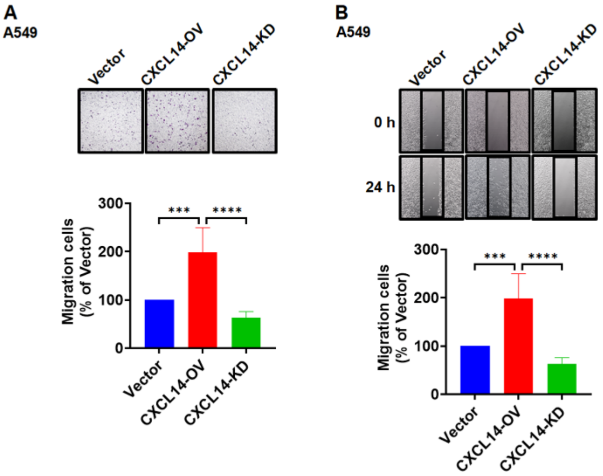

Supplementary Figure S5. CXCL14 regulates migration in lung cancer cells. (A, B) Cell migration of CXCL14-OV and CXCL14-KD A549 cells was measured with migration and wound healing assays (n = 4). Cells transfected with vector were used as controls.
